# Hypothalamic sex-specific metabolic shift by canagliflozin during aging

**DOI:** 10.1007/s11357-024-01214-z

**Published:** 2024-05-27

**Authors:** Hashan S. M. Jayarathne, Ryan Sullivan, Lukas Stilgenbauer, Lucas K. Debarba, Artur Kuchumov, Lisa Koshko, Sydney Scofield, Wanqing Liu, Brett C. Ginsburg, Richard A. Miller, Marianna Sadagurski

**Affiliations:** 1 Department of Biological Sciences, Integrative Biosciences Center, Wayne State University, Room 2418 IBio, 6135 Woodward, Detroit, MI 48202 USA; 2 Department of Pharmaceutical Science, Wayne State University, Detroit, MI USA; 3 Institute of Environmental Health Sciences, iBio (Integrative Biosciences Center), Wayne State University, Detroit, MI USA; 4 Department of Psychiatry and Behavioral Sciences, University of Texas Health Science Center, San Antonio, TX USA; 5 Department of Pathology, University of Michigan, Ann Arbor, MI USA

**Keywords:** Canagliflozin, Brain, Hypothalamus, Metabolism, Longevity

## Abstract

The hypothalamus undergoes significant changes with aging and plays crucial roles in age-related metabolic alterations. Sodium-glucose co-transporter 2 inhibitors (SGLT2i) are anti-diabetic agents that promote glucose excretion, and metabolic homeostasis. Recent studies have shown that a SGLT2i, Canagliflozin (Cana), can extend the median survival of genetically heterogeneous UM-HET3 male mice and improve central metabolic control via increases in hypothalamic insulin responsiveness in aged males, as well as reduced age-associated hypothalamic inflammation. We studied the long- and short-term effects of Cana on hypothalamic metabolic control in UM-HET3 mice. Starting the treatment from 7 months of age, we show that 4 weeks of Cana treatment significantly reduced body weight and fat mass in male but not female mice that was associated with enhanced glucose tolerance and insulin sensitivity observed by 12 months. Indirect calorimetry showed that Cana treatment increased energy expenditure in male, but not female mice, at 12 months of age. Long-term Cana treatment increased metabolic rates in both sexes, and markedly increasing formation of both orexigenic and anorexigenic projections to the paraventricular nucleus of the hypothalamus (PVH) mostly in females by 25 months. Hypothalamic RNA-sequencing analysis revealed increased sex-specific genes and signaling pathways related to insulin signaling, glycogen catabolic pathway, neuropeptide signaling, and mitochondrial function upregulated by Cana, with males showing a more pronounced and sustained effect on metabolic pathways at both age groups. Overall, our data provide critical evidence for sex-specific mechanisms that are affected by Cana during aging suggesting key targets of hypothalamic Cana-induced neuroprotection for metabolic control.

**Supplementary Information:**

The online version contains supplementary material available at 10.1007/s11357-024-01214-z.

## Introduction

The aging process is accompanied by various physiological changes, including alterations in hypothalamic function. The hypothalamus is a crucial brain region that serves as a master regulator of energy expenditure, body temperature, sleep–wake cycles, hunger and satiety, thirst, and hormone secretion [39]. Age-related changes in the hypothalamus can lead to a cascade of physiological dysfunctions that contribute to the onset and progression of metabolic diseases [2].

Emerging evidence suggests that anti-aging pharmacological interventions have the potential to improve whole-body metabolic health, perhaps by exerting beneficial effects on the hypothalamus during the aging process [21]. Sodium-glucose cotransporter 2 inhibitors (SGLT2i) were initially developed to manage T2D by reducing blood glucose levels independent of insulin. Notably, their efficacy in controlling blood glucose levels appears consistent between men and women [36]. Recent studies show that their beneficial effects extend beyond glycemic control [40, 42]. We have recently shown that an SGLT2i, Canagliflozin (Cana), extends the median survival of male mice by 14%, and robustly retards age-related lesions in male mice without corresponding effects on females [23, 32]. Moreover, long-term Cana treatment led to significantly lower fasting blood glucose levels and improved glucose tolerance by 22 months of age in both males and females [23]. SGLT2i cross the blood–brain barrier (BBB), and co-transporters are present in various parts of the brain [13, 35]. Indeed, treatment of pre-diabetic patients with the SGLT2i empagliflozin, for 8 weeks, restored hypothalamic insulin sensitivity, showing the beneficial effects of SGLT2i [15]. The beneficial effects of SGLT2i on overall metabolic homeostasis appear to require intact brain-periphery cross-talk via the parasympathetic nervous system [29].

We have previously demonstrated that at least some of the Cana effects on the hypothalamus of aged mice are sex-specific. For example, Cana treatment significantly improved insulin responsiveness in the hypothalamus of aged male but not female mice. Furthermore, Cana treatment improved exploratory and locomotor activity of 30-month-old male but not female mice. However, both male and female mice showed significant reductions in age-associated hypothalamic inflammation after Cana treatment [12]. The molecular mechanisms underlying the sex-specific neuroprotective effects of Cana on hypothalamic regulation of metabolism during aging are unclear.

In this study, we dissected the effects of Cana treatment on hypothalamic transcriptional remodeling during aging, using genetically heterogeneous UM-HET3 mice to avoid the genotype-specific idiosyncrasies and to replicate to some degree the variability seen in the human population [1].

## Materials and methods

### Mouse husbandry

Mice were bred as the progeny of (BALB/cByJ x C57BL/6 J)F1 mothers (JAX #100009) and (C3H/HeJ x DBA/2 J)F1 fathers (JAX #100004), so that each mouse is genetically unique and a full sibling to all other mice with respect to segregating nuclear alleles [10]. Mice are housed at three males or four females per cage from weaning and are provided food (TestDiet 5LG6: 17.5% protein, 5.6% fat) and water ad libitum. Mice in the Cana group (5LG6 w/10% 180 ppm Cana) received this agent at 180 mg per kg of chow, from 7 months of age; and sacrificed at 12 or 25 months of age as previously described [23]. All mice were provided with water ad libitum and housed in temperature-controlled rooms (22^0^C) on a 12-h/12-h light–dark cycle. If animals exhibited any indication of illness or distress, the laboratory staff conferred with on-site veterinary staff immediately to recommend appropriate interventions. Anesthesia for euthanasia was by avertin. Health status checks were conducted regularly in the animal facility. All animal experiments were performed in accordance with NIH guidelines for Animal Care and Use, approved and overseen by Wayne State University Institutional Animal Care and Use Committee (IACUC).

### Indirect calorimetry

In order to assess VO_2_ consumption, VCO_2_ production, respiratory exchange ratio (RER) and heat production, mice were placed in PhenoMaster metabolic cages (PhenoMaster, TSE system, Germany #160407–03). Animals were individually housed during 12 h of light and dark cycle (Dark cycle: 6 PM – 6 AM; light cycle: 6 AM-6 PM). The mice were acclimatized for 48 h and data were collected for 72 h while food and water were provided ad libitum.

### Glucose tolerance test

For the glucose tolerance test (GTT), mice were fasted for 6 h and injected intraperitoneally with D-glucose at a dose of 2 g/kg⋅BW. Blood glucose levels were measured at basal state (0 min) and at 15, 30, 60, 90, and 120 min after injection. Blood glucose levels were measured at the indicated times via tail vein bleeding using a OneTouch glucometer. Blood insulin was determined on serum from tail vein bleeds using Insulin ELISA kit (Crystal Chem. Inc.).

### Perfusion and immunolabeling

Perfusion and immunolabeling were performed as previously described [19]. Briefly, mice were anesthetized and perfused using phosphate buffered saline (PBS) (pH 7.5) followed by 4% paraformaldehyde. Brains were post-fixed, dehydrated, and then sectioned coronally (30 μm) using a sliding microtome, followed by immunofluorescent analysis. For immunohistochemistry brain sections were washed with PBS six times, and then blocked with 0.3% Triton X-100 and 3% normal donkey serum in PBS for 2 h; then the staining was carried out with the following primary antibodies overnight: rabbit anti-AgRP (1:1000; Phoenix Pharmaceuticals Inc; Cat.No.H-003–53), rabbit anti α-MSH (1:1000; Phoenix Pharmaceuticals Inc; Cat. No. H-043–01), rabbit anti-CART (1:2000; Phoenix Pharmaceuticals Inc.; Cat. No. H-003–62). Brain sections were incubated with Alexa Fluor-conjugated secondary antibodies for 2 h (Invitrogen). Microscopic images of the stained sections were obtained with a Nikon 800 fluorescent microscope using Nikon imaging DS-R12 color cooled SCMOS, version 5.00.

### Hypothalamic RNA extraction

Male mice were sacrificed at ad libitum to harvest the brain and to isolate the hypothalamus. Hypothalamus samples were lysed with 0.75 ml of 2-mercaptoethanol-supplemented lysis buffer (PureLink® RNA Mini Kit #12183025). Following the homogenization, with 0.75 ml of 70% ethanol, samples were transferred to spin cartridges with the collection tubes. Samples were centrifuged at 12000 × g for 15 s at room temperature. After washing the samples three times with washing buffers, cartridges were centrifuged at 12000 × g for 1–2 min to dry the membrane with bound RNA. RNA was eluted using 15–20 ul of RNase-free water.

### RNA sequencing and data analysis

Hypothalamic RNA-Seq was performed at the WSU Genome Sciences Core. RNA concentration was determined by NanoDrop (ND-1000 UV–Vis Spectrophotometer) and quality was assessed using RNA ScreenTape on a 4200 TapeStation. RNA-seq libraries were prepared according to the QIAseq Stranded RNA Library Kits protocol before sequencing on a NovaSeq 6000 (2 × 50 bp). Hypothalamic RNA sequences were mapped to the mouse reference genome (GRCm38.90) using HISAT2 v.2.1.0.13 following the adapter trimming and quality checking. Quantification of gene expression was generated using HTSeq-counts v0.6.0. Males and females were analyzed separately and following a PCA analysis of the raw data clear outliers were removed. Significantly differentially expressed genes (DEGs) were generated using the R Studio package DESeq2. Statistical significance was calculated by adjusting the P values with the Benjamini-Hochberg’s false discovery rate (FDR) method. All the noncoding RNA was removed from the analysis. DEGs were used to identify the enriched pathways, both Gene Ontology (for Biological Processes (BP)) and KEGG enrichment pathways using Gene Set Enrichment Analysis (GSEA) (NIH DAVID Bioinformatics https://david.ncifcrf.gov/home.jsp) (p-value cut off < 0.05). Heatmaps, bubble plots, Venn diagrams and gene expression changes were plotted using SRplot (https://bioinformatics.com.cn/). All RNA-Seq data are available at the Sequence Read Archive (SRA) at NCBI under accession number PRJNA1089563.

### Statistical analysis

Results are expressed as mean ± standard error and were analyzed using Statistica software (version 13.5.0.17). Graphs were generated using GraphPad Prism software. Sample size is annotated within figure legends. All parameters were analyzed by two-way ANOVA using the general linear model function and a full factorial model, which included an effect of treatment, the effect of sex, and the interaction effect between sex and treatment followed by the Newman–Keuls post hoc test. The level of significance (α) was set at 5%.

## Results

### Age- and sex-dependent changes in energy homeostasis in response to Cana feeding

**Fig. 1:**
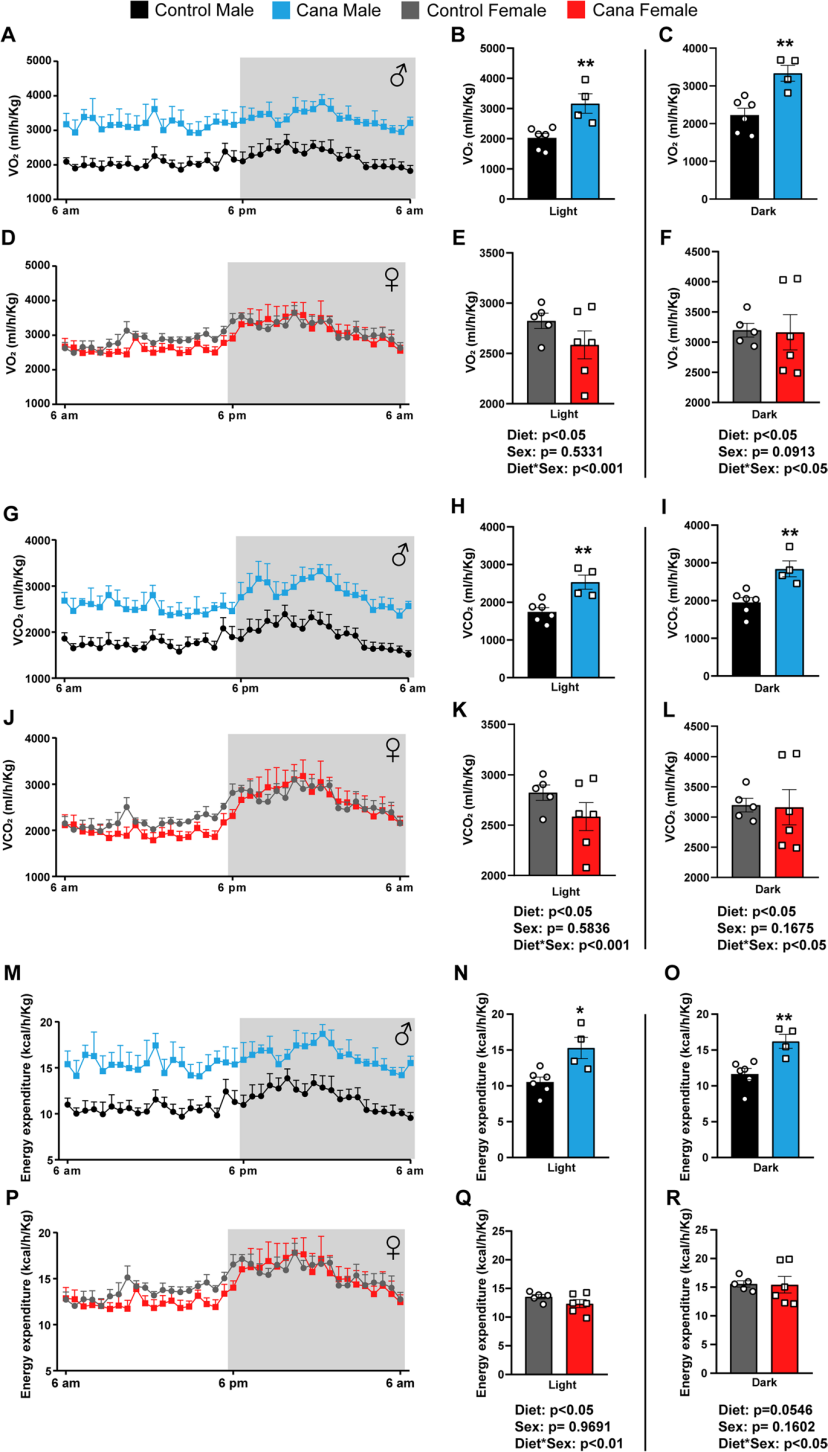
Cana feeding alters energy homeostasis parameters in 12-month-old male but not in female mice. **A**-**F** Oxygen consumption (VO_2_) in males (A-C) and females (D-F). **G**-**L** Carbon dioxide production (VCO_2_) in males (G-I) and females (J-L). **M**-**R** Energy expenditure in males (M–O) and females (P-R). Data represented as mean ± SEM, n = 5–6 mice/group. Two-way ANOVA followed by Newman-Keuls analysis (B-C, E–F, H-I, K-L, N–O and Q-R). *p < 0.05, **p < 0.01. P values for the effect of diet, sex and the interaction during light or dark cycle represent the significant p values from the two-way ANOVA

**Fig. 2:**
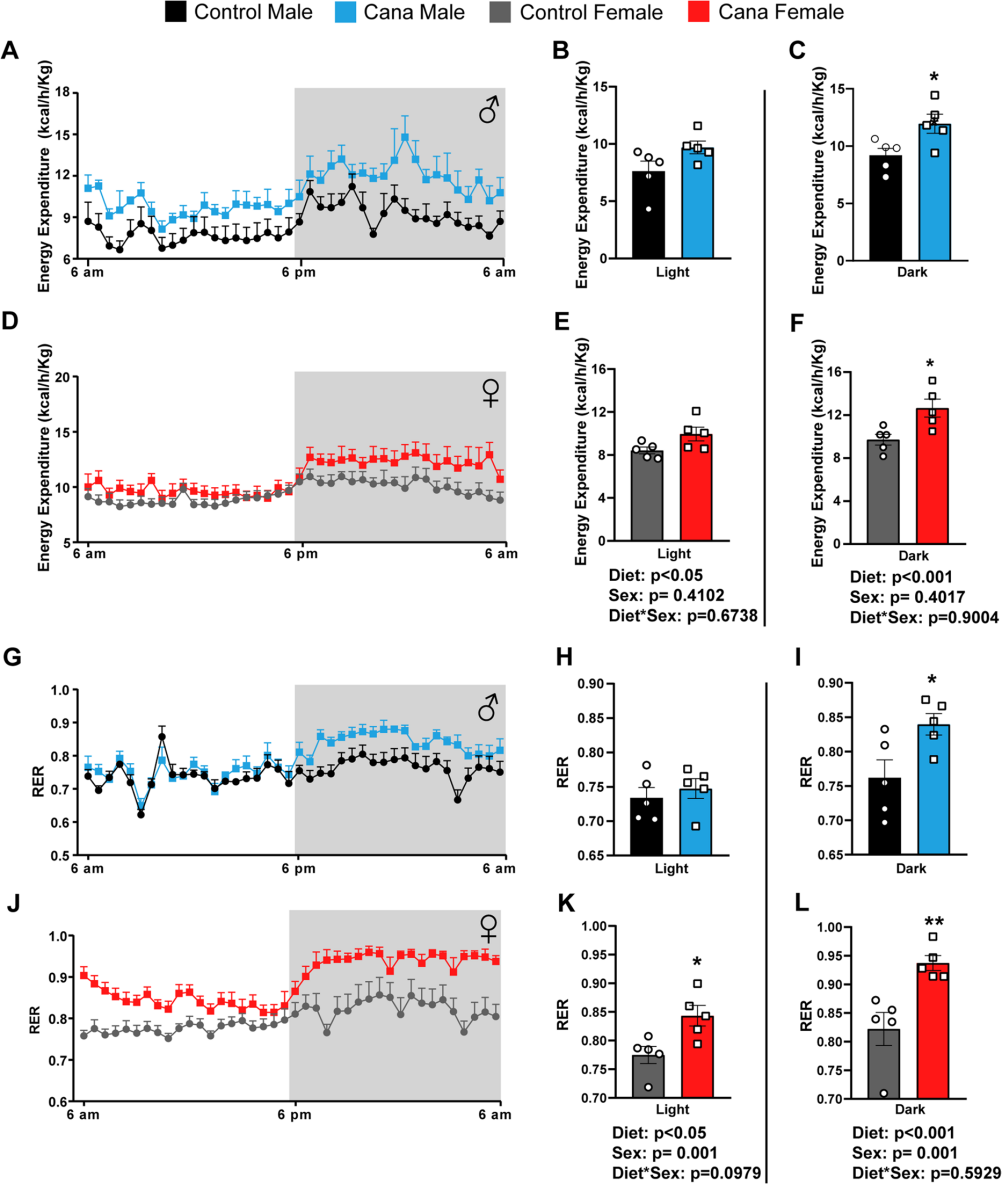
Cana feeding modulates energy homeostasis parameters in aged 25-month-old mice of both sexes. **A**-**F** Energy expenditure in males (A-C) and females (D-F). Respiratory exchange ratio (RER) in males (G-I) and females (J-L). Data represented as mean ± SEM, n = 5–6 mice/group. Two-way ANOVA followed by Newman-Keuls analysis (B-C, E–F, H-I and K-L). *p < 0.05, **p < 0.01. P values for the effect of diet, sex and the interaction during light or dark cycle represent the significant p values from the two-way ANOVA

We subjected 7-month-old genetically heterogeneous UM-HET3 mice to a Canagliflozin (Cana)-containing diet, as previously detailed [23]. After 4 weeks of treatment, Cana-fed male mice displayed a noticeable reduction in body weight and fat mass, while Cana-fed females did not show changes in body weight or body composition (Supplementary Fig. 1A-B). In our previous study we found that by the age of 12 months, Cana feeding led to a significant decrease in body weight in both sexes [23]. Both Cana-fed males and females exhibited slightly enhanced glucose tolerance, but only Cana-fed males showed increased insulin sensitivity, as evidenced by HOMA-IR (Supplementary Fig. 1C-F). To further assess the effect of Cana on metabolic control during aging, we measured parameters of whole-body energy homeostasis by indirect calorimetry. Oxygen consumption (VO_2_) and carbon dioxide production (VCO_2_) were significantly elevated throughout the dark and light cycle (p < 0.05 for effect of drug) only in Cana-fed 12-month-old males compared to females with a significant interaction between sex and drug treatment compared to control (p < 0.001 for light cycle, p < 0.05 for dark cycle) (Fig. 1A-L). Moreover, 12-month-old Cana-fed males exhibited a significant increase in energy expenditure (EE) during both light and dark cycles (p < 0.05 for effect of drug), without an effect on females, with a significant interaction between sex and drug treatment compared to control (p < 0.01 for light cycle, p < 0.05 for dark cycle) (Fig. 1M-R). There were no significant differences in respiratory exchange ratio (RER) or activity levels between controls or Cana-treated mice (Supplementary Fig. 2A-L). However, by 25 months of age, both aged Cana-fed male and female mice exhibited higher metabolic rate with increased EE and RER, particularly during the dark cycle (p < 0.001 for effect of drug) (Fig. 2 and Supplementary Fig. 3A-L). Cana-fed females demonstrated higher RER over the 24-h period (p < 0.01), indicative of an enhanced reliance on carbohydrates as fuel utilization. These changes in RER and energy expenditure were not associated with alterations in activity levels in 25-month-old Cana-fed mice (Supplementary Fig. 3M-R). 12- and 25-months-old Cana-fed mice exhibited significantly increased food consumption and water intake (p < 0.001 for effect of drug) (Supplementary Fig. 2M-P and 3S-V). Thus, prolonged Cana feeding supported improvement in energy homeostasis in aged mice of both sexes.

### Cana feeding alters hypothalamic orexigenic and anorexigenic projections in aged mice

**Fig. 3:**
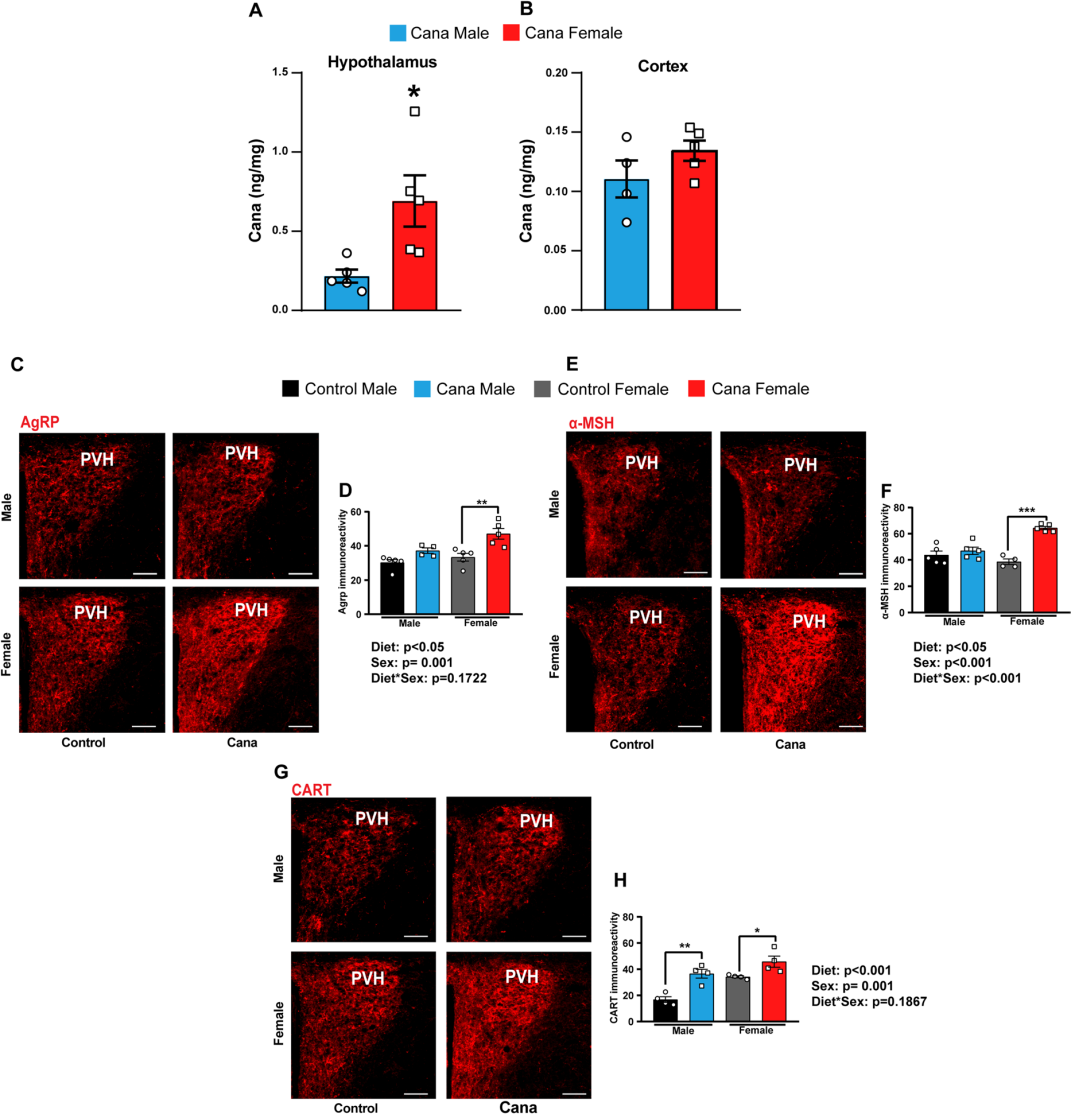
Cana feeding alters hypothalamic projections in aged 25-month-old mice. Cana levels measured in the hypothalamus (A) and cortex (B) in males (blue) and females (red), n = 4–5 mice/group. Images and quantification of (C-D) AgRP, (E–F) α-MSH and (G-H) CART immunoreactive fibers innervating the paraventricular nucleus of the hypothalamus (PVH) at 25 months of age in control and Cana treated male and female mice. Scale bar: 200 µm. Data represented as mean ± SEM, n = 4–5 mice/group. Two-way ANOVA followed by Newman-Keuls analysis (D, F and H). *p < 0.05, **p < 0.01, ***p < 0.001. P values for the effect of diet, sex and the diet x sex interaction represent the significant p values from the two-way ANOVA

Cana levels in the hypothalamus and cortex were measured using high-performance liquid chromatography/mass spectrometry as before [12]. In agreement with previous study [23], Cana levels in the hypothalamus of the females were significantly higher than males (0.36–1.25 ng/mg for females versus 0.12–0.38 ng/mg for males) (Fig. 3A), while Cana levels in the cortex were similar between males and females (Fig. 3B). To examine the effect of Cana on hypothalamic neuropeptides following treatment, we analyzed the immunoreactivity of agouti-related peptide (AgRP), alpha-melanocyte stimulating hormone (α-MSH), a product of the proopiomelanocortin (POMC), and cocaine- and amphetamine-regulated peptide (CART) containing fibers in the paraventricular nucleus (PVH) of the hypothalamus in 25-month-old mice. The density of orexigenic AgRP-IR fibers notably increased in the PVH of Cana-treated females, but not males, as compared to controls (p < 0.05 for effect of drug, p = 0.001 for effect of sex) (Fig. 3C and D). Additionally, the fiber density of anorexigenic α-MSH-IR was higher in Cana-treated females, but not males (p < 0.05 for effect of drug), with a significant interaction between sex and drug treatment (p < 0.001) (Fig. 3E and F). Interestingly, we observed significant upregulation in CART-IR in both Cana-treated males and females as compared to control mice (p < 0.001 for effect of drug) (Fig. 3G and H). To explore the possible involvement of the estrogen receptor α (ERα) in the sex-specific effects of Cana, we investigated its expression in the hypothalamus. As shown in Supplementary Fig. 4, while ERα expression was higher in aged females than in males (p < 0.01 for effect of sex), Cana treatment induced a comparable increase in ERα expression in both male and female mice (p < 0.001 for effect of drug).

### Cana feeding modulates the hypothalamic transcriptome

**Fig. 4:**
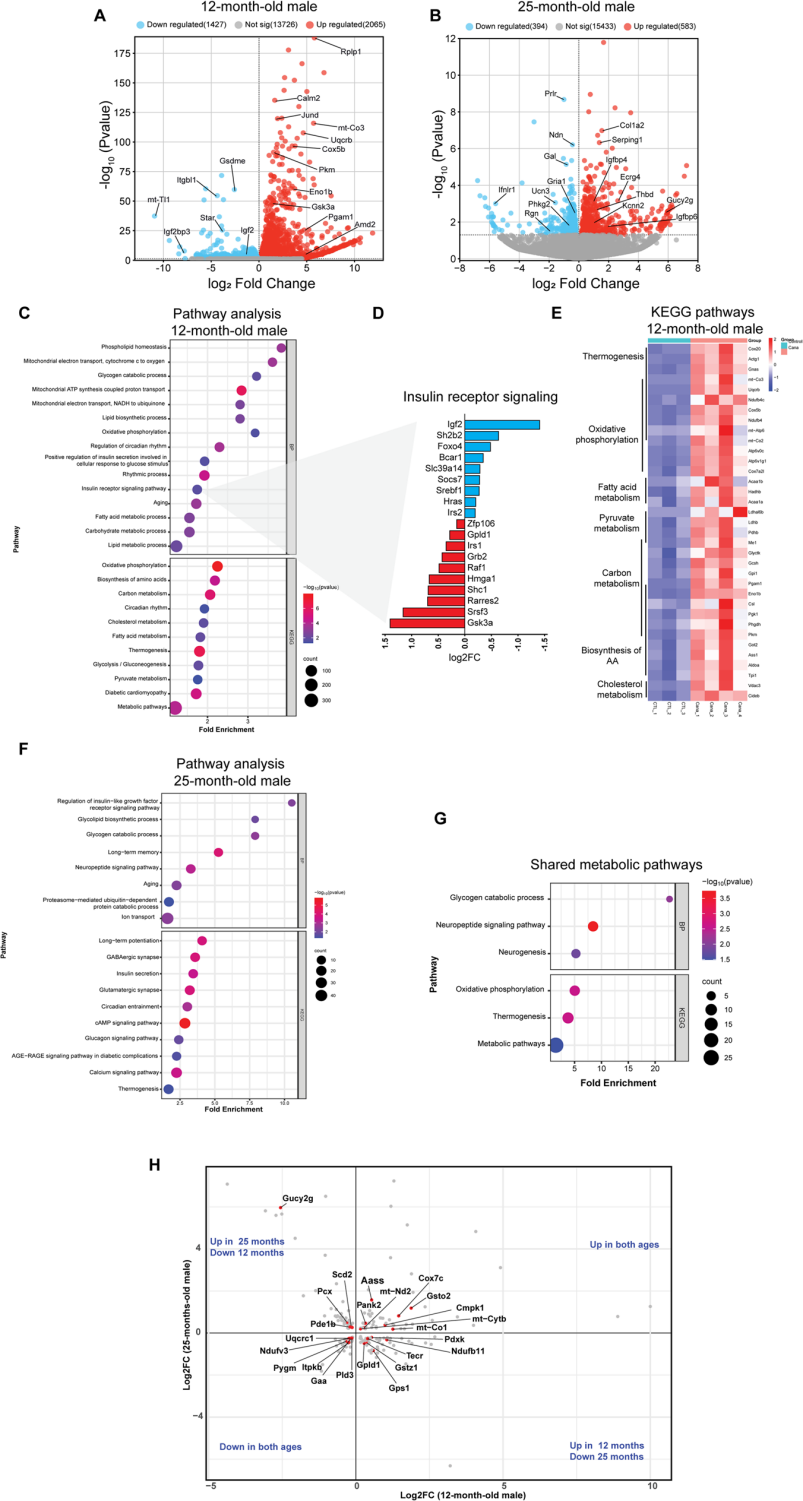
Modulation of hypothalamic transcriptome by Cana in male mice at 12 and 25-months. Volcano plots display differentially expressed genes (DEG, p < 0.05), with upregulated genes in red and downregulated genes in blue. **A** Represents 12-month-old, and (B) 25-month-old Cana versus control male mice. **C** Gene ontology (GO) analysis for biological processes (BP) and KEGG pathway analysis in 12-month-old Cana males. **D** Genes related to the BP "Insulin receptor signaling" significantly up and downregulated in 12-month-old Cana males. **E** Heatmap of top upregulated genes in KEGG pathways for 12-month-old Cana males. n = 3–4 mice/group. **F** GO analysis for BP and KEGG pathway analysis for 25-month-old Cana males. **G** Enriched transcriptomic pathways shared between 12 and 25 month-old males. (H) Scatter plot for shared metabolic genes between 12 and 25-month Cana males

To gain further insight on hypothalamic responses to Cana during aging, we performed bulk RNA-seq of microdissected hypothalami from Cana-treated males and females at 12- and 25-months of age (Fig. 4). The principal component analysis (PCA) plots demonstrated a clear separation in the gene expression profiles between Cana-treated male and female mice in both age groups (Supplementary Fig. 5). We first analyzed the effect of age separately for males and females, and then investigated the effect of sex in response to Cana.

*Hypothalamic transcriptome in male mice.* The volcano plots display genes upregulated and downregulated by Cana in the hypothalamus of male mice in both ages (Fig. 4A and B). We identified 3492 differentially expressed genes (DEGs) in hypothalami of 12-months-old Cana-treated males and 977 DEGs in 25-months-old Cana-treated males, with 196 genes shared between both age groups. Using Gene Set Enrichment Analysis (GSEA), we analyzed the Gene Ontology (GO-BP) and Kyoto Encyclopedia of Genes and Genomes (KEGG) databases to identify biological processes regulated by DEGs. GSEA analysis revealed upregulation of GO-BP terms related to ‘phospholipid homeostasis’, ‘oxidative phosphorylation’, ‘circadian rhythm’, and ‘insulin receptor signaling’ in Cana-treated 12-month-old males (Fig. 4C and Supplementary file 1). Figure 4D illustrates top genes in insulin receptor signaling modified by Cana in 12-month-old males. These genes include downregulated *IGF2*, *Foxo4, Irs2*, and *Sh2b2*, and upregulated *Irs1, Shc1* and *Gsk3a* among the others (Fig. 4D). KEGG analysis showed significant increases in metabolic pathways including carbon metabolism, thermogenesis, oxidative phosphorylation, cholesterol metabolism, fatty acid metabolism, and pyruvate metabolism (Fig. 4E). Examining the effects of hypothalamic aging under Cana, we observed upregulation of metabolism related pathways such as ‘insulin/IGF1 signaling’, ‘glycogen catabolic pathway’, ‘long-term memory’, ‘neuropeptide signaling’ and ‘thermogenesis’ among others in 25-month-old males (Fig. 4F and Supplementary file 1). Notably, GSEA of top overlapping hub genes as showed by scatter plot revealed shared enrichment of metabolic pathways such as ‘glycogen catabolic pathway’, ‘neuropeptide signaling’, ‘oxidative phosphorylation’, and ‘thermogenesis’ in males treated with Cana, for both age groups (Fig. 4G and H). These metabolic pathways commonly modified by Cana suggest their contribution to Cana-mediated hypothalamic traits related to metabolism in male mice.

**Fig. 5:**
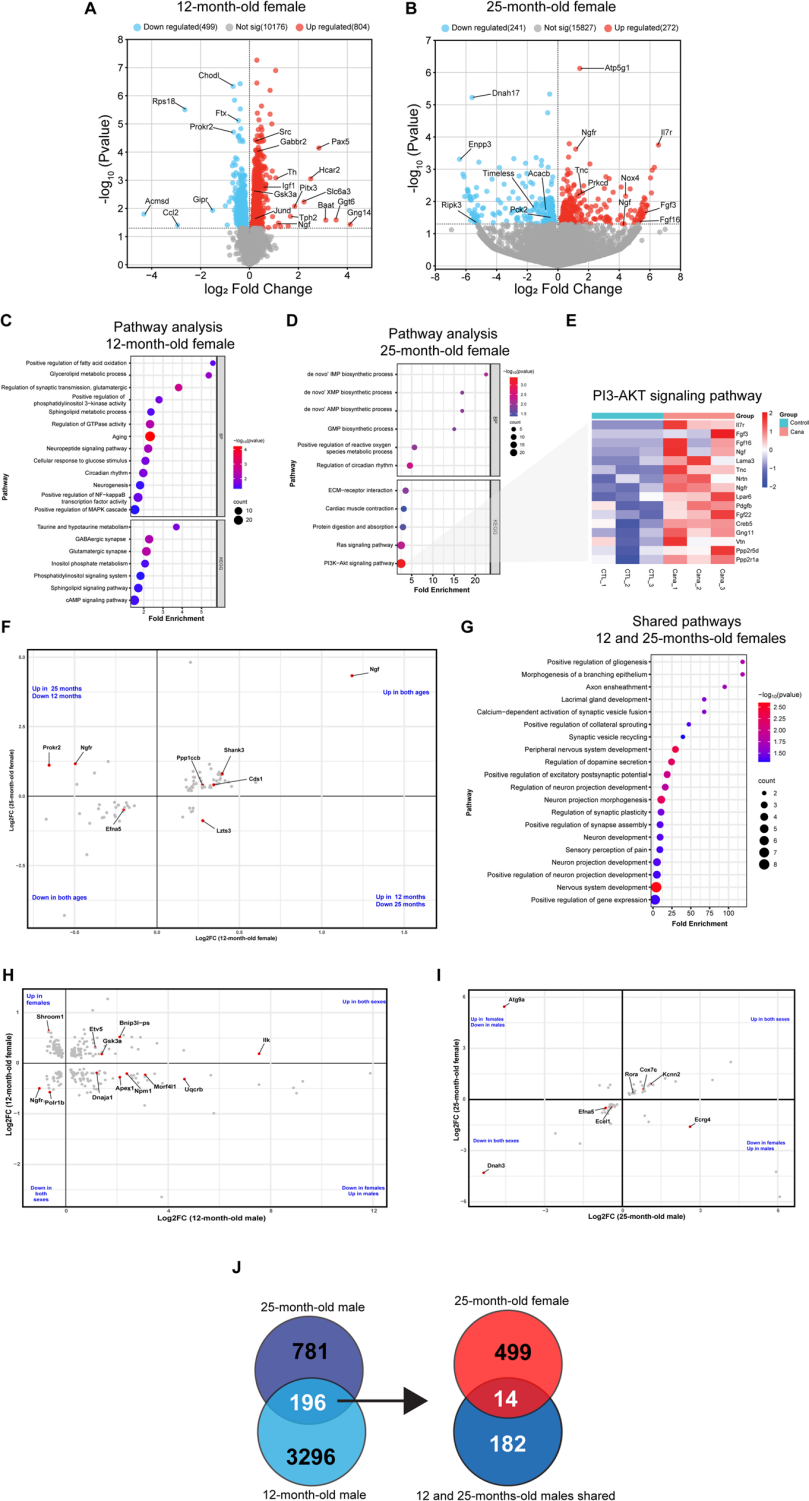
Modulation of hypothalamic transcriptome by Cana in male and female mice at 12 and 25-months. Volcano plots display differentially expressed genes (DEG, p < 0.05), with upregulated genes in red and downregulated genes in blue. **A** Represents 12-month-old, and (B) 25-month-old Cana versus control female mice. Gene ontology (GO) analysis for biological processes (BP) and KEGG pathway analysis in (C) 12-month-old and (D) 25-month-old Cana females. **E** Heatmap of top upregulated genes in “PI3-Akt signaling pathway” for 25-month-old Cana females. **F** Scatter plot for shared genes between 12 and 25-month Cana females. **G** Enriched transcriptomic pathways shared between 12 and 25 month-old females. n = 3–5 mice/group. Scatter plot for shared genes between (H) 12-month-old and (I) 25-month-old Cana males and females. **J** Venn diagram depicting the shared genes between Cana-treated males and 25-month-old females

*Hypothalamic transcriptome in female mice.* Figure 5A and B illustrate volcano plots displaying the upregulated and downregulated genes induced by Cana in the hypothalamus of female mice for 12 and 25 months of age. We identified altered genes enriched in GO terms in response to Cana treatment in female mice in both age groups. In 12-month-old females (Fig. 5C), these included terms related to metabolic processes such as 'positive regulation of fatty acid activation' and 'positive regulation of PI3-kinase activity', as well as pathways associated with ‘aging’, ‘circadian rhythms’, and ‘neurogenesis’. In contrast, 25-month-old females (Fig. 5D) exhibited enrichment in GO terms related to oxidative stress ('positive regulation of reactive oxygen species metabolic process'), as well as signaling pathways such as 'Ras signaling' and 'PI3K-Akt signaling pathway'. A heatmap illustrates the top genes in the PI3K-Akt signaling pathway affected by Cana treatment in 25-month-old females (Fig. 5E). These genes including upregulated *Fgf22, Creb5, IL7, Tnc, Ngfr and Pdgfb* possess broad cell survival activities. Supplementary file 2 shows the complete GO term and KEGG terms for females. When clustering the top genes with shared enrichment that were affected by Cana in females for both age groups, the clustered genes were primarily related to cellular function and development (Fig. 5F and G). Notably, no overlapping genes between the 12-month and 25-month-old females related to metabolic pathways were identified, indicating distinct age-related changes in female’s hypothalamic transcriptome affected by Cana.

*Effect of sex in response to Cana.* Finally, we explored the interaction between genes that were differentially expressed by sex in response to Cana. When comparing the transcriptomic changes of the hypothalamus upon Cana treatment in 12-month-old males and females, we observed no shared pathways between males and females, with only a few genes in these pathways exhibiting similar up- or downregulation. For example, the strongest upregulated shared genes *Gsk3a* and *Bnip3l-ps* were related to the ‘insulin signaling’ and ‘mitophagy’ pathways (Fig. 5H). Comparing the transcriptomic changes of the hypothalamus in 25-month-old males and females, only a few genes in shared pathways including ‘thermogenesis’ (*Cox7c*)’ and ‘neuropeptide signaling’ (*Ecrg4* and *Ecel1*) were similarly up- or downregulated (Fig. 5I). Moreover, among the limited number of genes (14 genes) shared among males in both age groups and 25-month-old females, a subset were identified to be related to mitochondrial function and metabolic regulation, such as *Cox7c, Hbb-bs, Gal3st3, Oip5os1,* and *Golga4*, suggesting their specific role in response to Cana (Fig. 5J). Supplementary file 3 shows the complete DEG set for both ages and sex.

## Discussion

The present study highlights the age- and sex-dependent effects of Cana treatment on hypothalamic function during aging, with males demonstrating significant metabolic improvements early on compared to females. Accordingly, Cana-treated males experience reductions in body weight and body adiposity sooner than Cana-treated females, which may explain the differences observed in their energy expenditure. Additionally, transcriptomic analysis delineates distinct Cana-responsive pathways in each sex, indicating different regulatory mechanisms governing hypothalamic traits related to metabolism in response to Cana during aging.

Part of the anti-aging effects of SGLT2is relates to their neuroprotective effects [8, 12, 20, 26]. In support, our current results highlight the effectiveness of Cana in the hypothalamus in mitigating age-related metabolic dysregulation in male mice. The early-onset and sustained improvements in metabolic parameters, including reduced body weight, fat mass, and enhanced glucose tolerance in aged UM-HET3 mice of both sexes, are consistent with the known mechanisms of action of Cana in improving peripheral insulin sensitivity and glucose metabolism [29, 42]. The significant alterations in hypothalamic gene expression observed in Cana-treated males in both age groups, particularly related to insulin signaling and neuropeptide signaling, align with our observations that Cana significantly improved central insulin sensitivity in the hypothalamus of aged male but not female mice [15]. Since hypothalamic insulin signaling plays a critical role in regulating energy homeostasis through modulation in neuropeptides signaling pathways [4, 33, 41], this may explain the robust effects of Cana on energy expenditure in male mice. On the other hand, female mice exhibited a delayed response to Cana treatment, with energy homeostasis improvements becoming evident only by 25 months of age. This delayed response in females may reflect sex-specific mechanistic differences observed in Cana mice, as well as differences in response to Cana, given that Cana levels are higher in the females compared to males, at any age. These findings underscore the importance of further investigating the underlying mechanisms driving these sex-dependent effects.

In aged mice, there is a significant reduction in AgRP and POMC neuropeptide neuronal activity [43], while the age-related metabolic dysfunction can be ameliorated by restoring the POMC gene in the arcuate nucleus (ARC) [18]. Although no genes related to metabolic pathways were identified in 25-month-old females, Cana treatment notably increased the density of AgRP and α-MSH-containing fibers exclusively in female mice compared to controls. Conversely, both Cana-treated male and female mice exhibited higher densities of anorexigenic CART-containing fibers in the PVH, accompanied by increased food and water intake. This supports the concept that SGLT2is induce weight loss partly through urinary calorie excretion, while simultaneously influencing central reward and satiety circuits, leading to increased appetite and food intake [38]. While both CART and α-MSH neurons are involved in regulating food intake and energy balance, they exhibit distinct patterns of activation and regulation [17]. An increase in both orexigenic and anorexigenic neuronal projections may indicate a compensatory response to metabolic alterations. For instance, during conditions of energy excess, such as obesity, increased AgRP projections may reflect an attempt to stimulate feeding behavior and restore energy balance. Conversely, elevated α-MSH/CART projections could signify an effort to enhance satiety signals and increase energy expenditure to counteract weight gain [28]. Thus, the augmentation of hypothalamic orexigenic and anorexigenic projections suggests a dynamic adaptation to metabolic changes aimed at restoring homeostasis. These neurons may further exhibit distinct sensitivities to the pharmacological effects of Cana, leading to differential alterations in their responsiveness, suggesting complex sex-specific responses to Cana treatment in the regulation of appetite and energy balance within the hypothalamus.

Sexual differences in responses to Cana might be attributed to differences in Cana pharmacodynamics because we detected a significant effect of sex in Cana levels, with females having higher Cana levels in the hypothalamus, and in the hippocampus, compared to males [12]. These data are consistent with previous report showing higher levels of Cana in the whole brain of females compared to males [23]. It is plausible that males and females metabolize Cana differently, leading to distinct pharmacokinetic profiles and responses to treatment. The observed sex-specific effects of Cana in the hypothalamus might suggest that Cana produces benefits through interaction with a metabolic or physiological pathway particularly relevant to male hypothalamus, or that Cana produced some side effects within the hypothalamus, directly or indirectly, in females. If the former hypothesis holds true, higher levels of Cana in aged females might potentially lead to some negative effects later in life as it accumulates in brain cells. These effects could counterbalance the benefits observed in younger female mice. Interestingly, a better metabolic response to SGLT2i was reported in men than in women [3, 6]. Further, sex differences in *Sglt2* were previously observed in various tissues with males expressing significantly higher levels of *Sglt2* in the whole brain, compared to females [24]. Previous studies have demonstrated that Cana exerts clinically relevant inhibitory effects on *Sglt1* at therapeutic plasma concentrations [27]. Further, there is evidence suggesting that combined SGLT1/2 inhibition might offer better control over glucose homeostasis compared to SGLT2 inhibitors alone [7]. Thus drug effect can be attributed to expression of *Sglt2* and *Sglt1* in different cell types and regions in the mouse brain. Further studies are needed to determine Cana pharmacological effects on hypothalamic function of both sexes, and to determine whether its accumulation poses a risk to female brain health at various stages of life.

In our previous study, we highlighted the impact of Cana on the hypothalamic inflammation, showing significant reductions in age-associated hypothalamic gliosis in 30-month-old male and female mice [12]. This improvement in hypothalamic inflammation may underlie the improvements in the metabolic homeostasis [11]. In the current study, we provide a comprehensive analysis of the hypothalamic transcriptome in response to Cana treatment which reveals sex-dependent molecular alterations underlying Cana metabolic impact in aging. Cana treatment elicits distinct gene expression profiles in male and female mice, with notable differences observed between age groups. In male mice, Cana induces significant upregulation of metabolic pathways, including phospholipid homeostasis, oxidative phosphorylation, and insulin receptor signaling, at both 12 and 25 months of age. These findings suggest a sustained metabolic response to Cana treatment and underscore the age-independent effects of Cana on hypothalamic gene expression in male mice. Conversely, female mice exhibit a less pronounced response to Cana treatment, with fewer differentially expressed genes identified compared to males. While Cana induces alterations in pathways related to fatty acid metabolism, PI3K-Akt signaling, and neuropeptide signaling in females, the overall response was weaker and less consistent in gene clusters across age groups. The comparison of conserved genes among males at both age groups and aged 25-month-old females, despite exhibiting phenotypically similar metabolic effects on energy homeostasis, revealed only a limited set of genes with roles in various aspects of cellular metabolism [5, 9, 30, 31, 37, 44]. Cana-treated males showed changes in pathways related to aging at 12 and 25 months of age while females showed changes only at 12 months of age. Notably, there were no pathways or genes related to the "aging" pathways in females by 25 months of age. Among the genes in this category that might be of specific interest in male mice are those related to insulin/IGF1 signaling and mitochondrial function, such as *Igf2, Irs2, Foxo4, Igfbp2, Gsk3a,* and *mt-Co1*. The downregulation of Irs2 is particularly noteworthy given its involvement in health and lifespan regulation in male mice [34]. This data suggests potential mechanisms responsible for sex-specific effects on longevity in Cana mice, highlighting the importance of insulin/IGF1 signaling and mitochondrial function in mediating Cana's effects on aging, especially in males. These findings further suggest that while aged males and females may exhibit similar metabolic responses to Cana treatment, the underlying central regulatory mechanisms and pathways driving these responses differ.

The metabolic responses to Cana share some similarities with those observed with caloric restriction (CR). Both interventions have been associated with improvements in glucose homeostasis, weight loss, and reductions in cardiovascular risk factors such as blood pressure and lipid levels [16]. CR typically leads to a reduction in circulating glucose and insulin levels, improved insulin sensitivity, and increased reliance on alternative energy sources such as fatty acids and ketones [25]. Similarly, Cana promotes glycosuria, leading to a decrease in plasma glucose levels and a subsequent increase in ketone production due to enhanced lipolysis and fatty acid oxidation [14]. Moreover, in the hypothalamus, CR induced the upregulation of genes involved in mitochondrial biogenesis, oxidative metabolism, and energy homeostasis [22], paralleling the effects induced by Cana in males. These adaptations may contribute to improved metabolic flexibility and energy utilization that we observed in Cana aged mice.

Overall, our study provides insights into the age- and sex-dependent effects of Cana treatment on hypothalamic traits related to metabolism. By revealing distinct sex-specific hypothalamic transcriptomic profiles in aged mice, we demonstrate the complexity of Cana's influence on energy homeostasis regulation in aging. Understanding the central sex-specific mechanisms behind these differences will not only enhance our understanding of metabolic regulation but also be essential for developing targeted therapies that optimize healthspan and promote healthy aging in both sexes.

### Supplementary Information

Below is the link to the electronic supplementary material.

Supplementary file1 (DOCX 8.95 MB)

Supplementary file2 (XLSX 129 KB)

Supplementary file3 (XLSX 84 KB)

Supplementary file4 (XLSX 11 KB)

## Data Availability

Further information and requests for resources and reagents should be directed to and will be fulfilled by the Lead Contact, Marianna Sadagurski (sadagurski@wayne.edu). Any additional information required to reanalyze the data reported in this paper is available from the lead contact upon request.

## Abbreviations

Cana: Canagliflozin
CNS: Central nervous system
SGLT2i: Sodium-glucose co-transporter 2 inhibitors
ARC: Arcuate nucleus of the hypothalamus
PVH: Paraventricular nucleus of the hypothalamus
