# Supplementary material for: Hypothalamic sex-specific metabolic shift by canagliflozin during aging
Source: GeroScience. 2024 May 27;46(5):4479–93. doi: 10.1007/s11357-024-01214-z (PMC11335982; doi:10.1007/s11357-024-01214-z)

**Supplementary Material**

**Hypothalamic Sex-Specific Metabolic Shift by Canagliflozin during Aging**

^1^Hashan S.M. Jayarathne, ^1^Ryan Sullivan, ^1^Lukas Stilgenbauer, ^1^Lucas K. Debarba, ^1^Artur Kuchumov, ^1^Lisa Koshko, ^1^Sydney Scofield, ^2^Wanqing Liu, ^4^Brett C. Ginsburg, ^5^Richard A. Miller, ^1,3^Marianna Sadagurski

1. Department of Biological Sciences, Wayne State University, Detroit, MI, USA

2. Department of Pharmaceutical Science, Wayne State University, Detroit, MI, USA

3. Institute of Environmental Health Sciences, iBio (Integrative Biosciences Center), Wayne State University, Detroit, MI, USA

4. Department of Psychiatry and Behavioral Sciences, University of Texas Health Science Center, San Antonio, TX, USA

5. Department of Pathology, University of Michigan, Ann Arbor, MI, USA

Running title: Cana effect on hypothalamus in aging

**Keywords:** Canagliflozin, brain, hypothalamus, metabolism, longevity

*Corresponding author:

Marianna Sadagurski,

Department of Biological Sciences,

Integrative Biosciences Center

Wayne State University

Room 2418 IBio,

6135 Woodward, Detroit, MI 48202, USA

Phone: (313) 577 8637

Email: [sadagurski@wayne.edu](mailto:sadagurski@wayne.edu)

**Supplementary Material**

**Supplementary Fig. 1: Body composition and glucose metabolism in Cana-fed mice.** Body weight (A) and fat mass (B) after 4 weeks of Cana treatment. HOMA IR at 12 months of age for males (C) and females (D). Glucose tolerance test at 12 months of age in males (E) and females (F). Data represented as mean ± SEM, n= 4-6 mice/group. Two-way ANOVA followed by Newman-Keuls analysis (B-C, E-F, H-I, K-L, N-O, and Q-R). *p<0.05, **p<0.01, ***p<0.001. P values for the effect of diet, sex, and the interaction represent the significant p values from the two-way ANOVA.

**Supplementary Fig. 2: Energy homeostasis parameters measured in 12-month-old Cana-fed mice.** (A-F) Respiratory exchange ratio (RER) for (A-C) males and (D-F) females. (G-L) Locomotor activity in (G-I) males and (J-L) females. Food intake in (M) males and (N) females. Water intake in males (O) and females (P). Data represented as mean ± SEM, n= 5-6 mice/group. Two-way ANOVA followed by Newman-Keuls analysis (B-C, E-F, H-I, K-L, N-O, and Q-R). **p<0.01. P values for the effect of diet, sex, and the interaction during 24 hours represent the significant p values from the two-way ANOVA.

**Supplementary Fig. 3: Energy homeostasis parameters measured in 25-month-old Cana-fed mice.** (A-F) Oxygen consumption (VO2) in males (A-C) and females (D-F). (G-L) Carbon dioxide production (VCO2) in males (G-I) and females (J-L). (M-R) Locomotor activity in males (M-O) and females (P-R). Food intake in males (S) and females (T). Water intake in males (U) and females (V). Data represented as mean ± SEM, n= 5-6 mice/group. Two-way ANOVA followed by Newman-Keuls analysis (B-C, E-F, H-I, K-L, N-O, and Q-R). *p<0.05, **p<0.01. P values for the effect of diet, sex, and the interaction between diet and sex during the light and dark cycle represent the significant p values from the two-way ANOVA.

**Supplementary Fig. 4:** Hypothalamic ER-α protein expression in Cana-treated mice. Brain sections of 25-mo-old male and female mice were analyzed for hypothalamic ER-α protein expression. (A) Representative images showing immunostaining in the arcuate nucleus of the hypothalamus (ARC) of control and Cana-treated mice. Scale bars: 200 μm. 3V, third ventricle. (B) Quantification of ER-α protein in the ARC from male and female mice; error bars show SEM for n = 4 mice of each type. Images were taken from at least three-four sections containing the hypothalamus. Data were analyzed by two-way ANOVA and further analyzed with the Newman–Keuls post hoc test (*p <0.05, ***p<0.001). P values for the effect of diet, sex, and the interaction between diet and sex represent the significant p values from the two-way ANOVA.

**Supplementary Fig. 5: Principal component analysis (PCA) of the hypothalamus isolated from Cana males and females.** Samples distribution of (A) 12-month-old male mice, (B) 12-month-old female mice, (C) 25-month-old male mice and (D) 25-month-old female mice. PCA was analyzed using the RStudio.Ink (version 2023.12.0369)

**Supplementary Fig. 1**


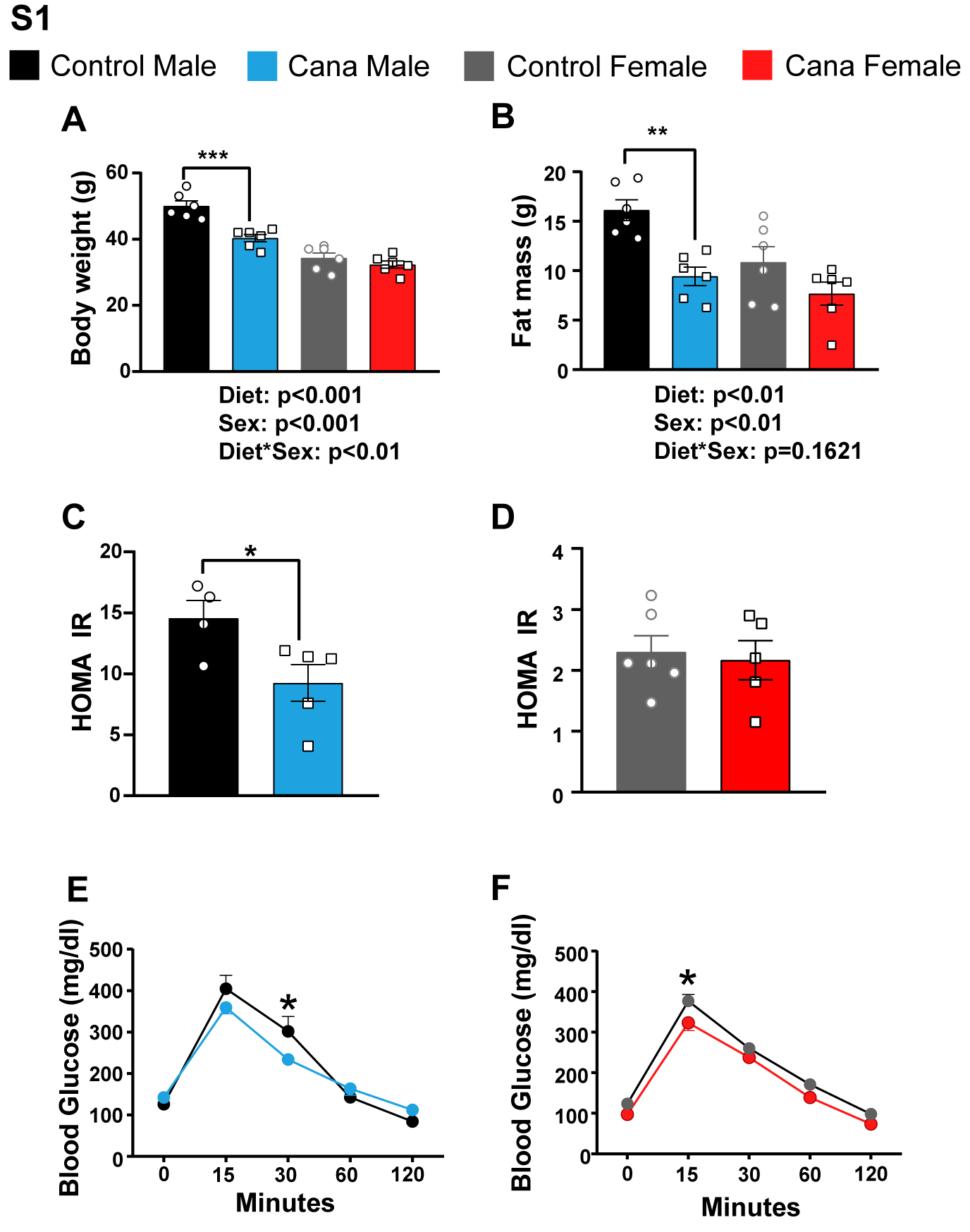


**Supplementary Fig. 2**


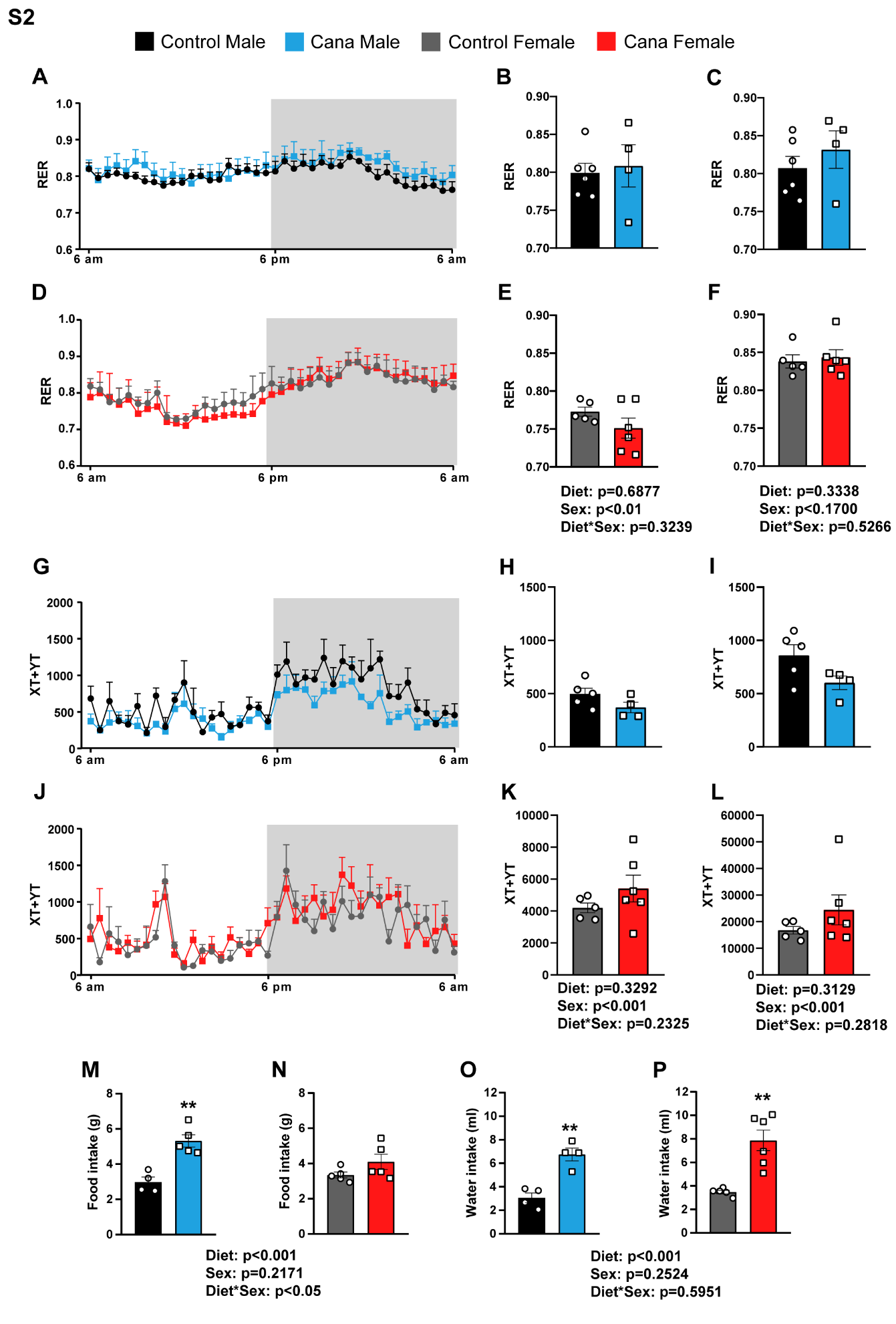


**
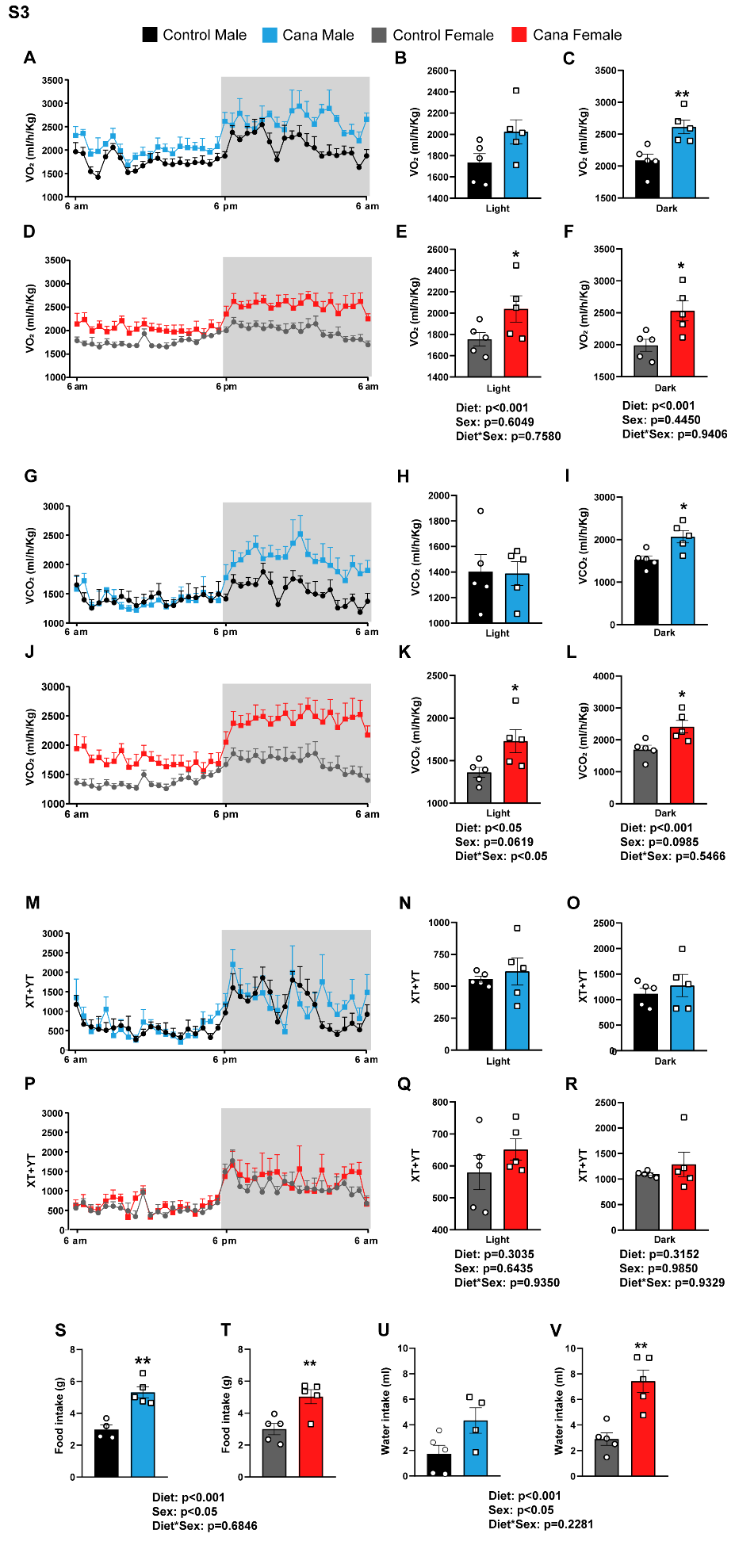
Supplementary Fig. 3**

**Supplementary Fig. 4**


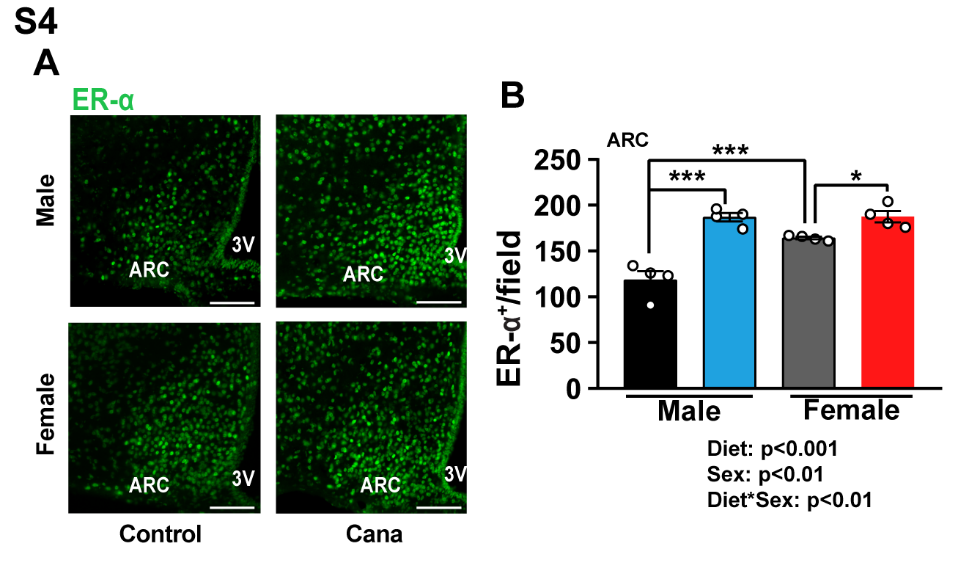


**Supplementary Fig. 5**
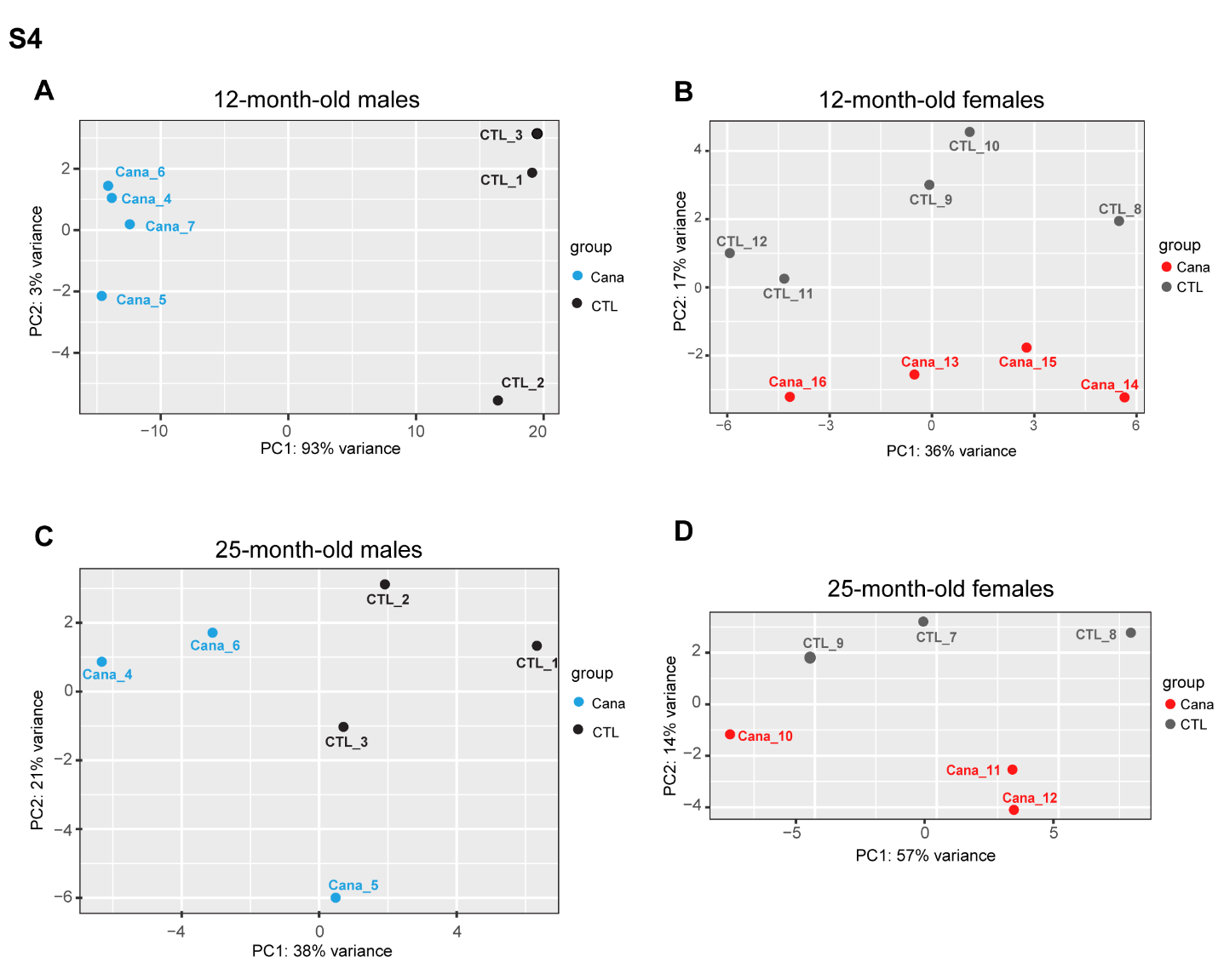

Supplement: Supplementary file 1 — Supplementary file1 (DOCX 8.95 MB) [file 11357_2024_1214_MOESM1_ESM.docx]
